# A triclinic polymorph of (*E*)-2-(4-iso­butyl­phen­yl)-*N*′-[1-(4-nitro­phen­yl)ethyl­idene]propano­hydrazide

**DOI:** 10.1107/S1600536813019892

**Published:** 2013-07-27

**Authors:** B. C. Manjunath, S. Madan Kumar, A. C. Vinayaka, S. Jayasheelan, M. P. Sadashiva, N. K. Lokanath

**Affiliations:** a Department of Studies in Physics, Manasagangotri, University of Mysore, Mysore, 570 006, India; b Department of Studies in Chemistry, Manasagangotri, University of Mysore, Mysore, 570 006, India; c Department of Physics, St Philomena’s College, Mysore, 570 006, India

## Abstract

The asymmetric unit of the triclinic polymorph of the title compound, C_21_H_25_N_3_O_3_, consists of two mol­ecules, whereas for the monoclinic polymorph Z′ = 1 [Fun *et al.* (2009 ▶). *Acta Cryst.* E**65**, o445]. The two mol­ecules exhibit an *E* configuration with respect to the C=N bond. The mol­ecules are linked into dimers by N—H⋯O and C—H⋯O hydrogen bonds forming *R*_2_^2^(8) ring motifs. In addition, π–π inter­actions occur between nitro­phenyl groups [minimum centroid–centroid distance 3.940 (2) Å], stacking the molecules along the *ac* plane.

## Related literature

For the structure of the monoclinic polymorph of the title compound, see: Fun *et al.* (2009 ▶). For graph-set notation, see: Bernstein *et al.* (1995 ▶). For the pharmacological activity of hydrazones, see: Bedia *et al.* (2006 ▶); Rollas *et al.* (2002 ▶); Terzioglu & Gursoy (2003 ▶).
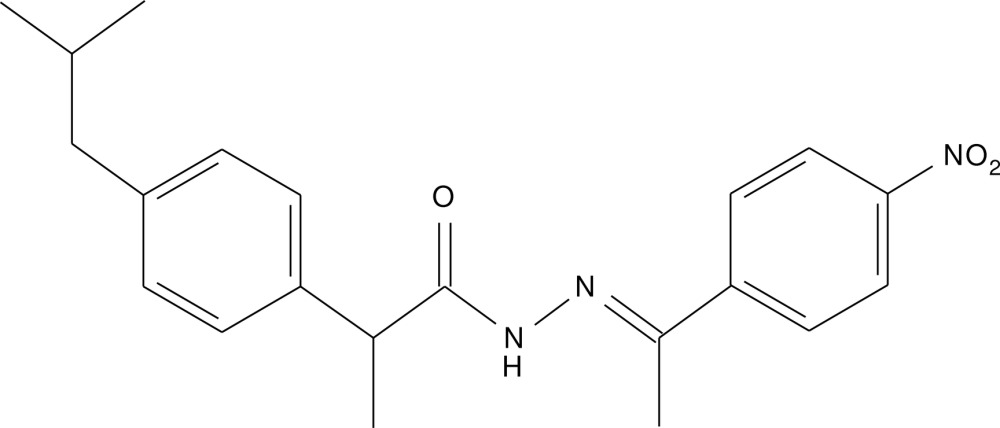


## Experimental

### 

#### Crystal data

C_21_H_25_N_3_O_3_
*M* *_r_* = 367.44
Triclinic, 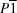

*a* = 12.201 (5) Å
*b* = 13.429 (5) Å
*c* = 13.932 (5) Å
α = 90.470 (7)°
β = 110.099 (6)°
γ = 107.321 (6)°
*V* = 2030.9 (13) Å^3^
*Z* = 4
Mo *K*α radiation
μ = 0.08 mm^−1^
*T* = 300 K
0.23 × 0.22 × 0.22 mm

#### Data collection

Oxford Diffraction Xcalibur Eos diffractometer
21740 measured reflections
8844 independent reflections
4673 reflections with *I* > 2σ(*I*)
*R*_int_ = 0.039

#### Refinement

*R*[*F* ^2^ > 2σ(*F* ^2^)] = 0.068
*wR*(*F* ^2^) = 0.193
*S* = 1.02
8844 reflections
495 parameters
H-atom parameters constrained
Δρ_max_ = 0.19 e Å^−3^
Δρ_min_ = −0.16 e Å^−3^

### 

Data collection: *CrysAlis PRO* (Oxford Diffraction, 2009 ▶); cell refinement: *CrysAlis PRO*; data reduction: *CrysAlis PRO*; program(s) used to solve structure: *SHELXS97* (Sheldrick, 2008 ▶); program(s) used to refine structure: *SHELXL97* (Sheldrick, 2008 ▶); molecular graphics: *Mercury* (Macrae *et al.*, 2008 ▶); software used to prepare material for publication: *SHELXL97*.

## Supplementary Material

Crystal structure: contains datablock(s) global, I. DOI: 10.1107/S1600536813019892/gk2579sup1.cif

Structure factors: contains datablock(s) I. DOI: 10.1107/S1600536813019892/gk2579Isup2.hkl

Supplementary material file. DOI: 10.1107/S1600536813019892/gk2579Isup3.cml

Additional supplementary materials:  crystallographic information; 3D view; checkCIF report

## Figures and Tables

**Table 1:** Hydrogen-bond geometry (Å, °)

*D*—H⋯*A*	*D*—H	H⋯*A*	*D*⋯*A*	*D*—H⋯*A*
N1*A*—H1*A*⋯O1*B* ^i^	0.86	2.14	2.977 (3)	165
N1*B*—H1*B*⋯O1*A* ^ii^	0.86	2.15	2.919 (3)	149
C15*A*—H15*C*⋯O1*B* ^i^	0.96	2.41	3.252 (4)	147

Symmetry codes: (i) 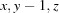; (ii) 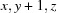.

**Fig. 1.**
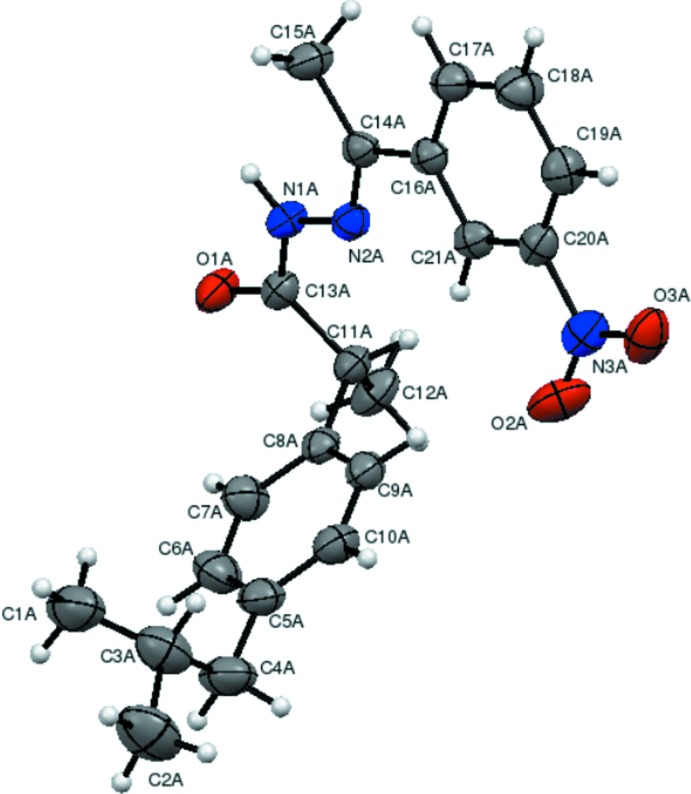
Molecule A of the title compound with displacement ellipsoids shown at the 50% probability level.

**Fig. 2.**
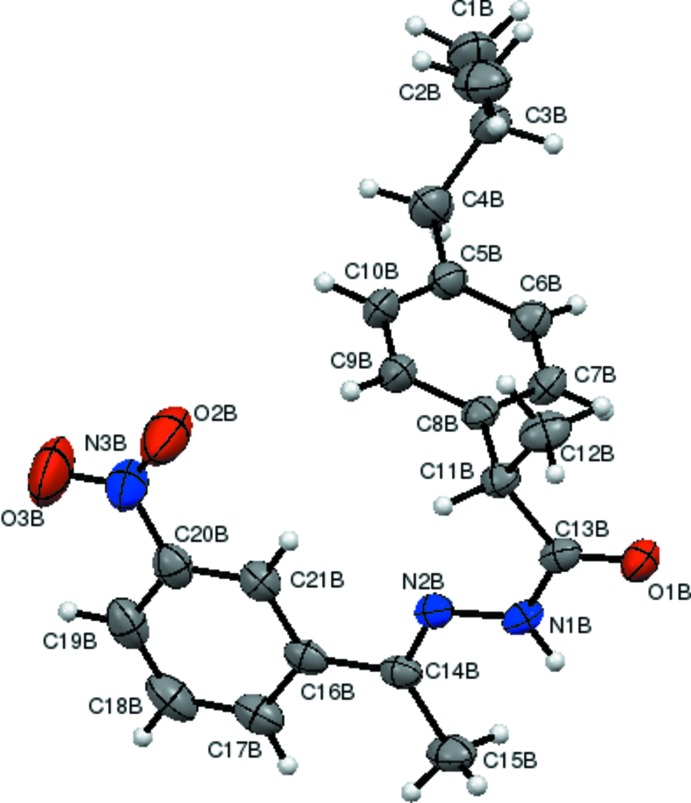
Molecule B of the title compound with displacement ellipsoids shown at the 50% probability level.

**Fig. 3.**
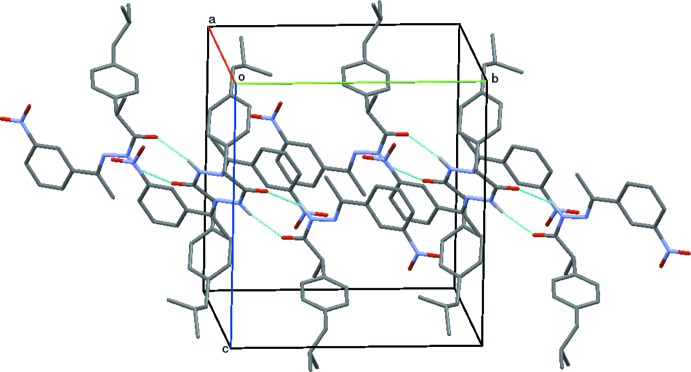
Packing diagram viewed along the crystallographic a axis. Dotted lines represent intermolecular N-H···O hydrogen bonding. Hydrogen atoms are not involved in the interactions were removed for clarity.

## supplementary crystallographic information

### Comment

Hydrazone derivatives show divers pharmacological activities (Bedia *et al.*, 2006; Rollas *et al.*, 2002; Terzioglu & Gursoy, 2003).

The asymmetric unit of the title compound consists of the *A* and *B* molecules (Fig. 1 & Fig. 2) and they show E configuration with respect to the C14=N2 bond. The molecular fragment composed of the atoms C11,C13,O1,N1,N2,C14,C15 is nearly planar, with the maximum deviation of 0.041 (3) Å for C15. It makes dihedral angles of 87.50 (14)°, 5.26 (14) ° and 42.43 (12) ° and 13.94 (12) ° with the terminal benzene rings in molecules B and A, respectively. The dihedral angle between nitrophenyl and phenyl groups are 87.64 (12)° and 74.31 ° for molecule *B* and *A*, respectively. These dihedral angles show that the two molecules differ in conformation. The bond lengths and bond angles are comparable to those in the monoclinic polymorph (Fun *et al.*, 2009).

The molecules are connected by N—H···O and C—H···O hydrogen bonds with *R*_2_^2^(8) ring motifs (Bernstein *et al.*, 1995) (Table 1 and Fig. 3). An intermolecular π···π interaction (*Cg*2 and *Cg*4; *Cg*4 and *Cg*4) is observed. The distance between *Cg*2 and *Cg*4 is 3.940 (3) Å and between *Cg*4 and *Cg*4 is 3.979 (3) Å. (*Cg*2 is C16*A*/C17*A*/C18*A*/C19*A*/C20*A*/C21*A* centroid and *Cg*4 is C16*B*/C17*B*/C18*B*/C19*B*/C20*B*/C21*B* centroid*)*. This interaction generates stacking of molecules along the *ac* plane.

### Experimental

The title compound is prepared by heating 2-(4-isobutylphenyl)propanehydrazide (0.01 mol) with *p*-nitroacetophenone(0.01 mol), in the presence of catalytic amount of acetic acid, in ethanol (20 ml) at reflux temperature for 5 h. Solid compound was obtained by filtration, washed with ice cold water and dried. The title compound was crystallized by slow evaporation of ethanol and acetonitrile (m.p. 442 K).

### Refinement

All the H atoms were placed in calculated positions, with N—H = 0.86 Å, *U_iso_* (H) = 1.2 *U_eq_*(N) for NH, C–H = 0.93 Å, *U_iso_* (H) = 1.2 *U_eq_*(C) for aromatic and C–H = 0.97 Å, *U*iso (H) = 1.2 *U_eq_*(C) for CH_2_, *U*iso (H) = 1.5 *U_eq_*(C) for CH_3_ atoms.

### Crystal data

**Table:**

C_21_H_25_N_3_O_3_	*Z* = 4
*M**_r_* = 367.44	*F*(000) = 784
Triclinic, *P*1	*D*_x_ = 1.202 Mg m^−^^3^
Hall symbol: -P 1	Melting point: 442 K
*a* = 12.201 (5) Å	Mo *K*α radiation, λ = 0.71073 Å
*b* = 13.429 (5) Å	Cell parameters from 8844 reflections
*c* = 13.932 (5) Å	θ = 1.6–27.1°
α = 90.470 (7)°	µ = 0.08 mm^−^^1^
β = 110.099 (6)°	*T* = 300 K
γ = 107.321 (6)°	Block, colorless
*V* = 2030.9 (13) Å^3^	0.23 × 0.22 × 0.22 mm

### Data collection

**Table:**

Oxford Diffraction Xcalibur Eos diffractometer	4673 reflections with *I* > 2σ(*I*)
Radiation source: fine-focus sealed tube	*R*_int_ = 0.039
Graphite monochromator	θ_max_ = 27.1°, θ_min_ = 1.6°
Detector resolution: 16.0839 pixels mm^-1^	*h* = −15→15
ω scans	*k* = −17→17
21740 measured reflections	*l* = −17→17
8844 independent reflections	

### Refinement

**Table:**

Refinement on *F*^2^	Primary atom site location: structure-invariant direct methods
Least-squares matrix: full	Secondary atom site location: difference Fourier map
*R*[*F*^2^ > 2σ(*F*^2^)] = 0.068	Hydrogen site location: inferred from neighbouring sites
*wR*(*F*^2^) = 0.193	H-atom parameters constrained
*S* = 1.02	*w* = 1/[σ^2^(*F*_o_^2^) + (0.0752*P*)^2^ + 0.3062*P*] where *P* = (*F*_o_^2^ + 2*F*_c_^2^)/3
8844 reflections	(Δ/σ)_max_ = 0.004
495 parameters	Δρ_max_ = 0.19 e Å^−^^3^
0 restraints	Δρ_min_ = −0.16 e Å^−^^3^

### Special details

**Table:**

Geometry. Bond distances, angles *etc*. have been calculated using the rounded fractional coordinates. All su's are estimated from the variances of the (full) variance-covariance matrix. The cell e.s.d.'s are taken into account in the estimation of distances, angles and torsion angles
Refinement. Refinement on *F*^2^ for ALL reflections except those flagged by the user for potential systematic errors. Weighted *R*-factors *wR* and all goodnesses of fit *S* are based on *F*^2^, conventional *R*-factors *R* are based on *F*, with *F* set to zero for negative *F*^2^. The observed criterion of *F*^2^ > σ(*F*^2^) is used only for calculating -*R*-factor-obs *etc*. and is not relevant to the choice of reflections for refinement. *R*-factors based on *F*^2^ are statistically about twice as large as those based on *F*, and *R*-factors based on ALL data will be even larger.

### Fractional atomic coordinates and isotropic or equivalent isotropic displacement parameters (Å^2^)

**Table:**

	*x*	*y*	*z*	*U*_iso_*/*U*_eq_	
O1A	0.84024 (16)	−0.14913 (15)	0.55714 (13)	0.0761 (7)	
O2A	0.7121 (2)	0.3312 (2)	0.70085 (17)	0.1031 (10)	
O3A	0.6171 (2)	0.44110 (17)	0.63494 (17)	0.0948 (9)	
N1A	0.71527 (17)	−0.05635 (16)	0.48633 (14)	0.0577 (7)	
N2A	0.67981 (18)	0.03078 (15)	0.49209 (14)	0.0551 (7)	
N3A	0.6410 (2)	0.3595 (2)	0.6291 (2)	0.0756 (10)	
C1A	0.6003 (4)	−0.1837 (3)	0.9456 (3)	0.1231 (18)	
C2A	0.6171 (4)	−0.0619 (4)	1.0906 (3)	0.134 (2)	
C3A	0.6444 (4)	−0.0725 (3)	0.9916 (3)	0.1029 (16)	
C4A	0.7774 (3)	−0.0140 (3)	1.0121 (2)	0.1044 (15)	
C5A	0.8123 (3)	−0.0023 (3)	0.9175 (2)	0.0768 (10)	
C6A	0.8563 (3)	−0.0728 (3)	0.8837 (2)	0.0910 (15)	
C7A	0.8846 (3)	−0.0651 (2)	0.7957 (2)	0.0797 (11)	
C8A	0.8700 (2)	0.0160 (2)	0.73670 (17)	0.0563 (8)	
C9A	0.8267 (2)	0.0874 (2)	0.77044 (19)	0.0639 (9)	
C10A	0.7972 (2)	0.0782 (2)	0.8581 (2)	0.0729 (10)	
C11A	0.9017 (2)	0.0263 (2)	0.64037 (18)	0.0592 (8)	
C12A	1.0355 (2)	0.0331 (2)	0.6613 (2)	0.0848 (11)	
C13A	0.8170 (2)	−0.0664 (2)	0.55925 (18)	0.0572 (9)	
C14A	0.5906 (2)	0.04170 (19)	0.41559 (17)	0.0530 (8)	
C15A	0.5237 (3)	−0.0325 (2)	0.31726 (19)	0.0773 (10)	
C16A	0.5552 (2)	0.13587 (19)	0.42770 (17)	0.0524 (8)	
C17A	0.4665 (2)	0.1624 (2)	0.34890 (19)	0.0698 (10)	
C18A	0.4373 (3)	0.2526 (3)	0.3600 (2)	0.0838 (11)	
C19A	0.4939 (3)	0.3181 (2)	0.4514 (2)	0.0733 (11)	
C20A	0.5797 (2)	0.2901 (2)	0.53021 (19)	0.0600 (9)	
C21A	0.6115 (2)	0.2023 (2)	0.52082 (17)	0.0561 (8)	
O1B	0.60116 (16)	0.75492 (14)	0.32931 (12)	0.0678 (6)	
O2B	0.7193 (3)	0.2616 (2)	0.2322 (2)	0.1500 (15)	
O3B	0.8201 (3)	0.1626 (2)	0.3011 (2)	0.1580 (16)	
N1B	0.70320 (18)	0.65361 (16)	0.42081 (14)	0.0575 (7)	
N2B	0.73709 (17)	0.56469 (16)	0.42424 (14)	0.0544 (7)	
N3B	0.7882 (3)	0.2379 (2)	0.3055 (2)	0.0912 (12)	
C1B	1.0431 (3)	0.6854 (3)	−0.0762 (3)	0.1043 (17)	
C2B	0.8173 (3)	0.6347 (3)	−0.1156 (2)	0.1057 (15)	
C3B	0.9435 (3)	0.6789 (2)	−0.0323 (2)	0.0760 (11)	
C4B	0.9575 (3)	0.6178 (3)	0.0603 (2)	0.0843 (12)	
C5B	0.8631 (3)	0.6109 (2)	0.10965 (18)	0.0642 (10)	
C6B	0.8514 (2)	0.6997 (2)	0.14981 (19)	0.0661 (10)	
C7B	0.7625 (2)	0.6943 (2)	0.19134 (17)	0.0601 (9)	
C8B	0.6818 (2)	0.59888 (19)	0.19621 (15)	0.0515 (8)	
C9B	0.6943 (3)	0.5099 (2)	0.15785 (18)	0.0674 (10)	
C10B	0.7823 (3)	0.5158 (2)	0.11506 (19)	0.0727 (11)	
C11B	0.5795 (2)	0.59323 (19)	0.23640 (16)	0.0552 (8)	
C12B	0.4704 (2)	0.6125 (2)	0.15221 (19)	0.0760 (10)	
C13B	0.6271 (2)	0.6736 (2)	0.33137 (17)	0.0544 (8)	
C14B	0.8072 (2)	0.5478 (2)	0.51048 (17)	0.0560 (8)	
C15B	0.8537 (3)	0.6172 (2)	0.61121 (18)	0.0788 (10)	
C16B	0.8429 (2)	0.4523 (2)	0.50529 (18)	0.0587 (9)	
C17B	0.9217 (2)	0.4223 (3)	0.5904 (2)	0.0757 (10)	
C18B	0.9561 (3)	0.3350 (3)	0.5827 (3)	0.0888 (14)	
C19B	0.9135 (3)	0.2737 (3)	0.4908 (3)	0.0844 (14)	
C20B	0.8343 (2)	0.3021 (2)	0.4058 (2)	0.0683 (10)	
C21B	0.7998 (2)	0.3889 (2)	0.41132 (19)	0.0616 (9)	
H1A	0.67210	−0.10530	0.43560	0.0690*	
H4A1	0.82740	−0.05020	1.05870	0.1250*	
H3A	0.59630	−0.03620	0.94160	0.1230*	
H4A2	0.79750	0.05550	1.04680	0.1250*	
H6A	0.86760	−0.12810	0.92150	0.1090*	
H7A	0.91390	−0.11510	0.77560	0.0960*	
H9A	0.81680	0.14350	0.73340	0.0770*	
H10A	0.76650	0.12740	0.87770	0.0880*	
H1A1	0.61870	−0.18750	0.88430	0.1850*	
H11A	0.88910	0.09070	0.61300	0.0710*	
H1A2	0.51290	−0.21200	0.92870	0.1850*	
H12D	1.04760	−0.03190	0.68230	0.1270*	
H12E	1.08950	0.08940	0.71510	0.1270*	
H12F	1.05330	0.04610	0.59980	0.1270*	
H1A3	0.64070	−0.22360	0.99420	0.1850*	
H2A1	0.66670	−0.09230	1.14350	0.2010*	
H2A2	0.53160	−0.09780	1.07700	0.2010*	
H15A	0.51060	0.00710	0.25970	0.1160*	
H15B	0.44560	−0.07640	0.31740	0.1160*	
H15C	0.57200	−0.07560	0.31190	0.1160*	
H2A3	0.63620	0.01110	1.11300	0.2010*	
H17A	0.42550	0.11830	0.28690	0.0840*	
H18A	0.37880	0.26920	0.30510	0.1010*	
H19A	0.47490	0.37890	0.45980	0.0880*	
H21A	0.67030	0.18670	0.57610	0.0670*	
H4B1	0.95220	0.54710	0.03880	0.1010*	
H1B	0.73030	0.69710	0.47570	0.0690*	
H4B2	1.03900	0.65070	0.11170	0.1010*	
H3B	0.95290	0.75090	−0.00840	0.0910*	
H2B1	0.81100	0.67760	−0.17080	0.1590*	
H2B2	0.75540	0.63420	−0.08760	0.1590*	
H6B	0.90500	0.76520	0.14880	0.0790*	
H2B3	0.80540	0.56430	−0.14140	0.1590*	
H7B	0.75670	0.75610	0.21660	0.0720*	
H1B1	1.02760	0.61810	−0.11160	0.1560*	
H1B2	1.12200	0.70520	−0.02120	0.1560*	
H9B	0.64230	0.44430	0.16080	0.0810*	
H1B3	1.04270	0.73700	−0.12350	0.1560*	
H10B	0.78740	0.45400	0.08910	0.0880*	
H11B	0.55160	0.52280	0.25590	0.0660*	
H12A	0.49630	0.68180	0.13330	0.1140*	
H12B	0.40510	0.60630	0.17770	0.1140*	
H12C	0.44150	0.56140	0.09300	0.1140*	
H15D	0.90770	0.68430	0.60720	0.1180*	
H15E	0.89770	0.58480	0.66580	0.1180*	
H15F	0.78510	0.62670	0.62470	0.1180*	
H17B	0.95180	0.46260	0.65410	0.0910*	
H18B	1.00910	0.31750	0.64090	0.1070*	
H19B	0.93680	0.21460	0.48530	0.1010*	
H21	0.74740	0.40610	0.35250	0.0740*	

### Atomic displacement parameters (Å^2^)

**Table:**

	*U*^11^	*U*^22^	*U*^33^	*U*^12^	*U*^13^	*U*^23^
O1A	0.0714 (12)	0.0606 (12)	0.0770 (12)	0.0225 (10)	0.0029 (9)	−0.0123 (9)
O2A	0.0921 (16)	0.131 (2)	0.0766 (14)	0.0391 (15)	0.0172 (12)	−0.0294 (13)
O3A	0.1093 (17)	0.0687 (15)	0.1118 (16)	0.0122 (13)	0.0610 (14)	−0.0153 (12)
N1A	0.0541 (12)	0.0579 (13)	0.0511 (11)	0.0133 (10)	0.0112 (9)	−0.0054 (9)
N2A	0.0577 (12)	0.0543 (13)	0.0531 (11)	0.0167 (10)	0.0210 (10)	0.0019 (9)
N3A	0.0684 (16)	0.0825 (19)	0.0784 (17)	0.0090 (14)	0.0431 (14)	−0.0097 (14)
C1A	0.120 (3)	0.147 (4)	0.077 (2)	0.010 (3)	0.033 (2)	0.014 (2)
C2A	0.175 (4)	0.166 (4)	0.109 (3)	0.078 (3)	0.090 (3)	0.040 (3)
C3A	0.119 (3)	0.125 (3)	0.084 (2)	0.053 (3)	0.048 (2)	0.029 (2)
C4A	0.112 (3)	0.131 (3)	0.0567 (17)	0.028 (2)	0.0239 (18)	0.0022 (18)
C5A	0.0759 (18)	0.088 (2)	0.0513 (15)	0.0187 (17)	0.0113 (13)	0.0007 (15)
C6A	0.112 (3)	0.094 (3)	0.0698 (19)	0.046 (2)	0.0249 (18)	0.0283 (17)
C7A	0.096 (2)	0.078 (2)	0.0737 (18)	0.0449 (18)	0.0266 (16)	0.0120 (16)
C8A	0.0483 (13)	0.0552 (16)	0.0519 (13)	0.0136 (12)	0.0047 (11)	−0.0004 (11)
C9A	0.0660 (16)	0.0578 (17)	0.0594 (15)	0.0199 (13)	0.0127 (13)	0.0023 (12)
C10A	0.0740 (18)	0.074 (2)	0.0630 (16)	0.0249 (15)	0.0150 (14)	−0.0074 (14)
C11A	0.0554 (14)	0.0537 (16)	0.0571 (14)	0.0101 (12)	0.0132 (11)	−0.0036 (11)
C12A	0.0590 (17)	0.084 (2)	0.091 (2)	0.0025 (15)	0.0206 (15)	−0.0202 (16)
C13A	0.0577 (15)	0.0531 (16)	0.0561 (14)	0.0139 (12)	0.0183 (12)	−0.0016 (11)
C14A	0.0516 (13)	0.0595 (16)	0.0462 (12)	0.0106 (12)	0.0218 (11)	0.0069 (11)
C15A	0.0811 (19)	0.082 (2)	0.0561 (15)	0.0279 (16)	0.0084 (13)	−0.0089 (13)
C16A	0.0488 (13)	0.0580 (15)	0.0504 (13)	0.0116 (11)	0.0227 (11)	0.0065 (11)
C17A	0.0737 (17)	0.074 (2)	0.0569 (15)	0.0262 (15)	0.0158 (13)	0.0056 (13)
C18A	0.092 (2)	0.086 (2)	0.0727 (18)	0.0438 (19)	0.0161 (16)	0.0094 (16)
C19A	0.0778 (19)	0.071 (2)	0.0837 (19)	0.0318 (16)	0.0378 (16)	0.0095 (15)
C20A	0.0568 (15)	0.0605 (17)	0.0660 (15)	0.0094 (13)	0.0343 (13)	−0.0024 (12)
C21A	0.0507 (13)	0.0659 (17)	0.0537 (13)	0.0143 (12)	0.0252 (11)	0.0047 (12)
O1B	0.0702 (11)	0.0641 (12)	0.0644 (10)	0.0252 (10)	0.0161 (9)	−0.0083 (9)
O2B	0.205 (3)	0.142 (3)	0.0968 (18)	0.106 (2)	0.005 (2)	−0.0231 (17)
O3B	0.201 (3)	0.116 (2)	0.168 (3)	0.101 (2)	0.038 (2)	−0.0118 (19)
N1B	0.0633 (12)	0.0630 (14)	0.0426 (10)	0.0180 (11)	0.0169 (9)	−0.0075 (9)
N2B	0.0570 (11)	0.0577 (13)	0.0471 (11)	0.0141 (10)	0.0210 (9)	0.0034 (9)
N3B	0.103 (2)	0.074 (2)	0.104 (2)	0.0396 (17)	0.0369 (18)	0.0064 (17)
C1B	0.135 (3)	0.093 (3)	0.117 (3)	0.038 (2)	0.083 (2)	0.017 (2)
C2B	0.118 (3)	0.118 (3)	0.0690 (19)	0.022 (2)	0.033 (2)	0.0003 (18)
C3B	0.100 (2)	0.0682 (19)	0.0791 (19)	0.0332 (17)	0.0504 (17)	0.0104 (15)
C4B	0.101 (2)	0.094 (2)	0.090 (2)	0.0529 (19)	0.0547 (18)	0.0229 (17)
C5B	0.0766 (17)	0.072 (2)	0.0564 (14)	0.0358 (16)	0.0289 (13)	0.0105 (13)
C6B	0.0628 (16)	0.0652 (18)	0.0709 (16)	0.0157 (13)	0.0292 (13)	0.0028 (13)
C7B	0.0598 (15)	0.0557 (17)	0.0620 (15)	0.0143 (13)	0.0228 (12)	−0.0090 (12)
C8B	0.0555 (14)	0.0536 (15)	0.0383 (11)	0.0134 (12)	0.0121 (10)	−0.0031 (10)
C9B	0.0882 (19)	0.0559 (17)	0.0632 (15)	0.0196 (14)	0.0366 (14)	0.0018 (12)
C10B	0.107 (2)	0.065 (2)	0.0667 (16)	0.0440 (18)	0.0423 (16)	0.0071 (13)
C11B	0.0551 (14)	0.0557 (16)	0.0476 (12)	0.0094 (12)	0.0174 (11)	−0.0043 (11)
C12B	0.0565 (15)	0.099 (2)	0.0598 (15)	0.0178 (15)	0.0123 (13)	−0.0072 (14)
C13B	0.0503 (13)	0.0615 (17)	0.0483 (13)	0.0097 (12)	0.0214 (11)	−0.0016 (11)
C14B	0.0555 (14)	0.0595 (16)	0.0464 (13)	0.0051 (12)	0.0220 (11)	0.0082 (11)
C15B	0.089 (2)	0.082 (2)	0.0462 (13)	0.0077 (16)	0.0183 (13)	0.0008 (13)
C16B	0.0507 (13)	0.0666 (18)	0.0540 (14)	0.0063 (12)	0.0235 (11)	0.0182 (12)
C17B	0.0678 (18)	0.088 (2)	0.0623 (16)	0.0152 (16)	0.0208 (14)	0.0247 (15)
C18B	0.076 (2)	0.104 (3)	0.086 (2)	0.034 (2)	0.0236 (17)	0.049 (2)
C19B	0.081 (2)	0.077 (2)	0.111 (3)	0.0325 (18)	0.047 (2)	0.042 (2)
C20B	0.0647 (16)	0.0629 (18)	0.0821 (19)	0.0182 (14)	0.0341 (15)	0.0219 (15)
C21B	0.0592 (15)	0.0637 (18)	0.0622 (15)	0.0196 (13)	0.0221 (12)	0.0158 (13)

### Geometric parameters (Å, º)

**Table:**

O1A—C13A	1.228 (3)	C12A—H12D	0.9600
O2A—N3A	1.226 (4)	C15A—H15A	0.9600
O3A—N3A	1.224 (4)	C15A—H15C	0.9600
O1B—C13B	1.222 (3)	C15A—H15B	0.9600
O2B—N3B	1.194 (4)	C17A—H17A	0.9300
O3B—N3B	1.195 (4)	C18A—H18A	0.9300
N1A—N2A	1.374 (3)	C19A—H19A	0.9300
N1A—C13A	1.351 (3)	C21A—H21A	0.9300
N2A—C14A	1.282 (3)	C1B—C3B	1.519 (6)
N3A—C20A	1.477 (4)	C2B—C3B	1.512 (5)
N1A—H1A	0.8600	C3B—C4B	1.522 (4)
N1B—C13B	1.359 (3)	C4B—C5B	1.513 (5)
N1B—N2B	1.371 (3)	C5B—C6B	1.379 (4)
N2B—C14B	1.281 (3)	C5B—C10B	1.383 (4)
N3B—C20B	1.471 (4)	C6B—C7B	1.379 (4)
N1B—H1B	0.8600	C7B—C8B	1.383 (4)
C1A—C3A	1.483 (6)	C8B—C11B	1.517 (4)
C2A—C3A	1.541 (6)	C8B—C9B	1.375 (4)
C3A—C4A	1.500 (6)	C9B—C10B	1.380 (5)
C4A—C5A	1.514 (4)	C11B—C13B	1.526 (3)
C5A—C10A	1.384 (4)	C11B—C12B	1.534 (4)
C5A—C6A	1.369 (5)	C14B—C15B	1.509 (3)
C6A—C7A	1.380 (4)	C14B—C16B	1.481 (4)
C7A—C8A	1.387 (4)	C16B—C21B	1.399 (4)
C8A—C11A	1.516 (3)	C16B—C17B	1.396 (4)
C8A—C9A	1.373 (4)	C17B—C18B	1.373 (5)
C9A—C10A	1.384 (4)	C18B—C19B	1.363 (6)
C11A—C12A	1.530 (4)	C19B—C20B	1.386 (5)
C11A—C13A	1.518 (4)	C20B—C21B	1.362 (4)
C14A—C16A	1.479 (4)	C1B—H1B1	0.9600
C14A—C15A	1.504 (3)	C1B—H1B2	0.9600
C16A—C17A	1.387 (4)	C1B—H1B3	0.9600
C16A—C21A	1.398 (3)	C2B—H2B1	0.9600
C17A—C18A	1.382 (5)	C2B—H2B2	0.9600
C18A—C19A	1.374 (4)	C2B—H2B3	0.9600
C19A—C20A	1.376 (4)	C3B—H3B	0.9800
C20A—C21A	1.365 (4)	C4B—H4B1	0.9700
C1A—H1A1	0.9600	C4B—H4B2	0.9700
C1A—H1A3	0.9600	C6B—H6B	0.9300
C1A—H1A2	0.9600	C7B—H7B	0.9300
C2A—H2A2	0.9600	C9B—H9B	0.9300
C2A—H2A3	0.9600	C10B—H10B	0.9300
C2A—H2A1	0.9600	C11B—H11B	0.9800
C3A—H3A	0.9800	C12B—H12A	0.9600
C4A—H4A2	0.9700	C12B—H12B	0.9600
C4A—H4A1	0.9700	C12B—H12C	0.9600
C6A—H6A	0.9300	C15B—H15D	0.9600
C7A—H7A	0.9300	C15B—H15E	0.9600
C9A—H9A	0.9300	C15B—H15F	0.9600
C10A—H10A	0.9300	C17B—H17B	0.9300
C11A—H11A	0.9800	C18B—H18B	0.9300
C12A—H12F	0.9600	C19B—H19B	0.9300
C12A—H12E	0.9600	C21B—H21	0.9300
			
N2A—N1A—C13A	121.0 (2)	C16A—C17A—H17A	119.00
N1A—N2A—C14A	118.38 (19)	C19A—C18A—H18A	120.00
O2A—N3A—O3A	123.9 (3)	C17A—C18A—H18A	120.00
O2A—N3A—C20A	117.8 (2)	C18A—C19A—H19A	121.00
O3A—N3A—C20A	118.3 (2)	C20A—C19A—H19A	121.00
C13A—N1A—H1A	119.00	C20A—C21A—H21A	120.00
N2A—N1A—H1A	120.00	C16A—C21A—H21A	120.00
N2B—N1B—C13B	120.56 (19)	C1B—C3B—C4B	112.1 (3)
N1B—N2B—C14B	118.6 (2)	C1B—C3B—C2B	110.4 (3)
O2B—N3B—C20B	119.3 (3)	C2B—C3B—C4B	112.2 (3)
O3B—N3B—C20B	118.3 (3)	C3B—C4B—C5B	114.8 (3)
O2B—N3B—O3B	122.5 (3)	C6B—C5B—C10B	116.4 (3)
C13B—N1B—H1B	120.00	C4B—C5B—C10B	122.1 (3)
N2B—N1B—H1B	120.00	C4B—C5B—C6B	121.6 (3)
C1A—C3A—C4A	115.7 (4)	C5B—C6B—C7B	121.9 (3)
C1A—C3A—C2A	111.7 (4)	C6B—C7B—C8B	121.4 (2)
C2A—C3A—C4A	109.5 (3)	C7B—C8B—C11B	121.3 (2)
C3A—C4A—C5A	115.1 (3)	C7B—C8B—C9B	117.0 (3)
C4A—C5A—C6A	122.0 (3)	C9B—C8B—C11B	121.6 (2)
C6A—C5A—C10A	116.2 (3)	C8B—C9B—C10B	121.4 (3)
C4A—C5A—C10A	121.8 (3)	C5B—C10B—C9B	121.9 (3)
C5A—C6A—C7A	122.6 (3)	C8B—C11B—C12B	110.17 (18)
C6A—C7A—C8A	121.0 (3)	C8B—C11B—C13B	110.2 (2)
C7A—C8A—C9A	116.7 (2)	C12B—C11B—C13B	110.4 (2)
C7A—C8A—C11A	121.8 (2)	O1B—C13B—N1B	119.5 (2)
C9A—C8A—C11A	121.5 (2)	N1B—C13B—C11B	117.8 (2)
C8A—C9A—C10A	121.8 (2)	O1B—C13B—C11B	122.7 (2)
C5A—C10A—C9A	121.7 (3)	N2B—C14B—C16B	114.5 (2)
C12A—C11A—C13A	109.0 (2)	C15B—C14B—C16B	120.5 (2)
C8A—C11A—C13A	110.1 (2)	N2B—C14B—C15B	125.0 (2)
C8A—C11A—C12A	112.6 (2)	C17B—C16B—C21B	116.9 (3)
O1A—C13A—N1A	119.5 (2)	C14B—C16B—C17B	123.1 (2)
N1A—C13A—C11A	118.9 (2)	C14B—C16B—C21B	120.0 (2)
O1A—C13A—C11A	121.6 (2)	C16B—C17B—C18B	121.8 (3)
C15A—C14A—C16A	119.7 (2)	C17B—C18B—C19B	120.8 (3)
N2A—C14A—C15A	124.7 (2)	C18B—C19B—C20B	118.0 (3)
N2A—C14A—C16A	115.7 (2)	N3B—C20B—C19B	119.0 (3)
C14A—C16A—C21A	120.4 (2)	N3B—C20B—C21B	118.7 (2)
C17A—C16A—C21A	117.3 (2)	C19B—C20B—C21B	122.3 (3)
C14A—C16A—C17A	122.3 (2)	C16B—C21B—C20B	120.2 (2)
C16A—C17A—C18A	121.8 (2)	C3B—C1B—H1B1	109.00
C17A—C18A—C19A	120.6 (3)	C3B—C1B—H1B2	109.00
C18A—C19A—C20A	117.4 (3)	C3B—C1B—H1B3	109.00
C19A—C20A—C21A	123.2 (2)	H1B1—C1B—H1B2	109.00
N3A—C20A—C21A	118.8 (2)	H1B1—C1B—H1B3	110.00
N3A—C20A—C19A	118.1 (2)	H1B2—C1B—H1B3	109.00
C16A—C21A—C20A	119.7 (2)	C3B—C2B—H2B1	109.00
C3A—C1A—H1A1	110.00	C3B—C2B—H2B2	109.00
C3A—C1A—H1A2	109.00	C3B—C2B—H2B3	109.00
H1A1—C1A—H1A2	109.00	H2B1—C2B—H2B2	109.00
H1A2—C1A—H1A3	109.00	H2B1—C2B—H2B3	109.00
H1A1—C1A—H1A3	110.00	H2B2—C2B—H2B3	110.00
C3A—C1A—H1A3	109.00	C1B—C3B—H3B	107.00
H2A1—C2A—H2A3	109.00	C2B—C3B—H3B	107.00
C3A—C2A—H2A1	109.00	C4B—C3B—H3B	107.00
C3A—C2A—H2A2	109.00	C3B—C4B—H4B1	108.00
C3A—C2A—H2A3	109.00	C3B—C4B—H4B2	109.00
H2A1—C2A—H2A2	110.00	C5B—C4B—H4B1	109.00
H2A2—C2A—H2A3	109.00	C5B—C4B—H4B2	109.00
C4A—C3A—H3A	106.00	H4B1—C4B—H4B2	108.00
C2A—C3A—H3A	106.00	C5B—C6B—H6B	119.00
C1A—C3A—H3A	106.00	C7B—C6B—H6B	119.00
C3A—C4A—H4A1	108.00	C6B—C7B—H7B	119.00
C3A—C4A—H4A2	109.00	C8B—C7B—H7B	119.00
C5A—C4A—H4A2	109.00	C8B—C9B—H9B	119.00
H4A1—C4A—H4A2	108.00	C10B—C9B—H9B	119.00
C5A—C4A—H4A1	108.00	C5B—C10B—H10B	119.00
C5A—C6A—H6A	119.00	C9B—C10B—H10B	119.00
C7A—C6A—H6A	119.00	C8B—C11B—H11B	109.00
C8A—C7A—H7A	119.00	C12B—C11B—H11B	109.00
C6A—C7A—H7A	120.00	C13B—C11B—H11B	109.00
C10A—C9A—H9A	119.00	C11B—C12B—H12A	109.00
C8A—C9A—H9A	119.00	C11B—C12B—H12B	109.00
C9A—C10A—H10A	119.00	C11B—C12B—H12C	109.00
C5A—C10A—H10A	119.00	H12A—C12B—H12B	109.00
C13A—C11A—H11A	108.00	H12A—C12B—H12C	110.00
C8A—C11A—H11A	108.00	H12B—C12B—H12C	109.00
C12A—C11A—H11A	108.00	C14B—C15B—H15D	109.00
H12D—C12A—H12F	110.00	C14B—C15B—H15E	109.00
C11A—C12A—H12E	109.00	C14B—C15B—H15F	109.00
H12D—C12A—H12E	109.00	H15D—C15B—H15E	109.00
C11A—C12A—H12D	109.00	H15D—C15B—H15F	109.00
H12E—C12A—H12F	110.00	H15E—C15B—H15F	109.00
C11A—C12A—H12F	109.00	C16B—C17B—H17B	119.00
H15A—C15A—H15C	110.00	C18B—C17B—H17B	119.00
H15B—C15A—H15C	109.00	C17B—C18B—H18B	120.00
C14A—C15A—H15B	109.00	C19B—C18B—H18B	120.00
C14A—C15A—H15C	110.00	C18B—C19B—H19B	121.00
C14A—C15A—H15A	109.00	C20B—C19B—H19B	121.00
H15A—C15A—H15B	109.00	C16B—C21B—H21	120.00
C18A—C17A—H17A	119.00	C20B—C21B—H21	120.00
			
C13A—N1A—N2A—C14A	172.6 (2)	C21A—C16A—C17A—C18A	2.2 (4)
N2A—N1A—C13A—O1A	173.5 (2)	C17A—C16A—C21A—C20A	−1.2 (4)
N2A—N1A—C13A—C11A	−8.1 (3)	C14A—C16A—C21A—C20A	178.7 (2)
N1A—N2A—C14A—C15A	−2.2 (4)	C16A—C17A—C18A—C19A	−1.6 (5)
N1A—N2A—C14A—C16A	178.7 (2)	C17A—C18A—C19A—C20A	0.0 (5)
O2A—N3A—C20A—C19A	175.9 (3)	C18A—C19A—C20A—N3A	−179.8 (3)
O2A—N3A—C20A—C21A	−4.8 (4)	C18A—C19A—C20A—C21A	1.0 (5)
O3A—N3A—C20A—C19A	−3.4 (4)	C19A—C20A—C21A—C16A	−0.4 (4)
O3A—N3A—C20A—C21A	175.9 (3)	N3A—C20A—C21A—C16A	−179.6 (2)
N2B—N1B—C13B—C11B	1.1 (4)	C1B—C3B—C4B—C5B	178.4 (3)
C13B—N1B—N2B—C14B	−178.8 (2)	C2B—C3B—C4B—C5B	−56.8 (4)
N2B—N1B—C13B—O1B	−177.7 (2)	C3B—C4B—C5B—C6B	−59.8 (4)
N1B—N2B—C14B—C15B	1.9 (4)	C3B—C4B—C5B—C10B	118.8 (3)
N1B—N2B—C14B—C16B	−177.5 (2)	C4B—C5B—C6B—C7B	177.5 (2)
O2B—N3B—C20B—C21B	−2.0 (5)	C10B—C5B—C6B—C7B	−1.2 (4)
O2B—N3B—C20B—C19B	179.5 (4)	C4B—C5B—C10B—C9B	−178.4 (3)
O3B—N3B—C20B—C21B	179.1 (3)	C6B—C5B—C10B—C9B	0.3 (4)
O3B—N3B—C20B—C19B	0.6 (5)	C5B—C6B—C7B—C8B	1.1 (4)
C2A—C3A—C4A—C5A	−170.2 (3)	C6B—C7B—C8B—C9B	0.0 (3)
C1A—C3A—C4A—C5A	62.6 (4)	C6B—C7B—C8B—C11B	−176.5 (2)
C3A—C4A—C5A—C6A	−92.9 (4)	C7B—C8B—C9B—C10B	−0.9 (4)
C3A—C4A—C5A—C10A	84.7 (4)	C11B—C8B—C9B—C10B	175.6 (2)
C4A—C5A—C6A—C7A	177.8 (3)	C7B—C8B—C11B—C12B	80.2 (3)
C10A—C5A—C6A—C7A	0.1 (5)	C7B—C8B—C11B—C13B	−41.9 (3)
C4A—C5A—C10A—C9A	−178.6 (3)	C9B—C8B—C11B—C12B	−96.2 (3)
C6A—C5A—C10A—C9A	−0.9 (5)	C9B—C8B—C11B—C13B	141.7 (2)
C5A—C6A—C7A—C8A	0.3 (5)	C8B—C9B—C10B—C5B	0.8 (4)
C6A—C7A—C8A—C9A	0.2 (4)	C8B—C11B—C13B—O1B	105.5 (3)
C6A—C7A—C8A—C11A	179.4 (3)	C8B—C11B—C13B—N1B	−73.2 (3)
C7A—C8A—C9A—C10A	−1.0 (4)	C12B—C11B—C13B—O1B	−16.4 (3)
C7A—C8A—C11A—C13A	64.8 (3)	C12B—C11B—C13B—N1B	164.9 (2)
C9A—C8A—C11A—C12A	122.2 (3)	N2B—C14B—C16B—C17B	178.0 (3)
C9A—C8A—C11A—C13A	−115.9 (3)	N2B—C14B—C16B—C21B	−0.3 (4)
C11A—C8A—C9A—C10A	179.8 (2)	C15B—C14B—C16B—C17B	−1.3 (4)
C7A—C8A—C11A—C12A	−57.0 (3)	C15B—C14B—C16B—C21B	−179.6 (3)
C8A—C9A—C10A—C5A	1.4 (4)	C14B—C16B—C17B—C18B	−178.1 (3)
C12A—C11A—C13A—O1A	37.8 (3)	C21B—C16B—C17B—C18B	0.3 (5)
C8A—C11A—C13A—O1A	−86.1 (3)	C14B—C16B—C21B—C20B	178.7 (2)
C8A—C11A—C13A—N1A	95.5 (3)	C17B—C16B—C21B—C20B	0.4 (4)
C12A—C11A—C13A—N1A	−140.6 (2)	C16B—C17B—C18B—C19B	−0.4 (6)
N2A—C14A—C16A—C21A	−4.6 (4)	C17B—C18B—C19B—C20B	−0.2 (6)
C15A—C14A—C16A—C17A	−3.9 (4)	C18B—C19B—C20B—N3B	179.3 (3)
N2A—C14A—C16A—C17A	175.3 (2)	C18B—C19B—C20B—C21B	0.9 (5)
C15A—C14A—C16A—C21A	176.2 (3)	N3B—C20B—C21B—C16B	−179.4 (3)
C14A—C16A—C17A—C18A	−177.7 (3)	C19B—C20B—C21B—C16B	−0.9 (4)

### Hydrogen-bond geometry (Å, º)

**Table:**

*D*—H···*A*	*D*—H	H···*A*	*D*···*A*	*D*—H···*A*
N1*A*—H1*A*···O1*B*^i^	0.86	2.14	2.977 (3)	165
N1*B*—H1*B*···O1*A*^ii^	0.86	2.15	2.919 (3)	149
C11*A*—H11*A*···N2*A*	0.98	2.41	2.803 (4)	103
C15*A*—H15*C*···O1*B*^i^	0.96	2.41	3.252 (4)	147
C15*A*—H15*C*···N1*A*	0.96	2.41	2.791 (4)	103

Symmetry codes: (i) *x*, *y*−1, *z*; (ii) *x*, *y*+1, *z*.
